# PET-PCR reveals low parasitaemia and submicroscopic malarial infections in Honduran Moskitia

**DOI:** 10.1186/s12936-023-04538-x

**Published:** 2023-03-28

**Authors:** Gabriela Matamoros, Denis Escobar, Alejandra Pinto, Delmy Serrano, Eliška Ksandrová, Nicole Grimaldi, Gabriel Juárez-Fontecha, Marcela Moncada, Hugo O. Valdivia, Gustavo Fontecha

**Affiliations:** 1 Microbiology Research Institute, Universidad Nacional Autónoma de Honduras, Tegucigalpa, Honduras; 2 Hospital de Puerto Lempira, Secretaría de Salud de Honduras, Gracias a Dios, Honduras; 3 Department of Parasitology, U.S. Naval Medical Research Unit 6 (NAMRU-6), 07006 Lima, Peru

**Keywords:** Submicroscopic malaria, Parasitaemia, *Plasmodium* species, Nested PCR, PET-PCR, Honduras

## Abstract

**Background:**

Malaria remains a main parasitic disease of humans. Although the largest number of cases is reported in the African region, there are still endemic foci in the Americas. Central America reported 36,000 malaria cases in 2020, which represents 5.5% of cases in the Americas and 0.015% of cases globally. Most malaria infections in Central America are reported in La Moskitia, shared by Honduras and Nicaragua. In the Honduran Moskitia, less than 800 cases were registered in 2020, considering it an area of low endemicity. In low endemicity settings, the number of submicroscopic and asymptomatic infections tends to increase, leaving many cases undetected and untreated. These reservoirs challenge national malaria elimination programmes. This study aimed to assess the diagnostic performance of Light Microscopy (LM), a nested PCR test and a photoinduced electron transfer polymerase chain reaction (PET-PCR) in a population of febrile patients from La Moskitia.

**Methods:**

A total of 309 febrile participants were recruited using a passive surveillance approach at the Puerto Lempira hospital. Blood samples were analysed by LM, nested PCR, and PET-PCR. Diagnostic performance including sensitivity, specificity, negative and positive predictive values, kappa index, accuracy, and ROC analysis was evaluated. The parasitaemia of the positive samples was quantified by both LM and PET-PCR.

**Results:**

The overall prevalence of malaria was 19.1% by LM, 27.8% by nPCR, and 31.1% by PET-PCR. The sensitivity of LM was 67.4% compared to nPCR, and the sensitivity of LM and nPCR was 59.6% and 80.8%, respectively, compared to PET-PCR. LM showed a kappa index of 0.67, with a moderate level of agreement. Forty positive cases by PET-PCR were not detected by LM.

**Conclusions:**

This study demonstrated that LM is unable to detect parasitaemia at low levels and that there is a high degree of submicroscopic infections in the Honduran Moskitia.

## Background

Malaria is still one of the most serious parasitic diseases affecting humans. *Plasmodium* spp. infections have left deep traces in the human genome as a result of co-evolution and natural selection [1], in addition to having greatly influenced a long list of historical milestones [1, 2], ranging from the expansion of the Roman Empire to the construction of the Panama canal [3, 4]. During the first two decades of the third millennium, the international community has made great strides in malaria control, with 23 countries now reporting three consecutive years with zero indigenous cases of malaria, and twelve countries certified malaria-free by the World Health Organization (WHO), three of them in the Americas (Argentina, Paraguay, and El Salvador) [5]. Despite the worrying increase in the number of cases observed in recent years in the mining region of Venezuela, the WHO Region of the Americas has reduced malaria by 58% between 2000 and 2020 [5].

**Fig. 1:**
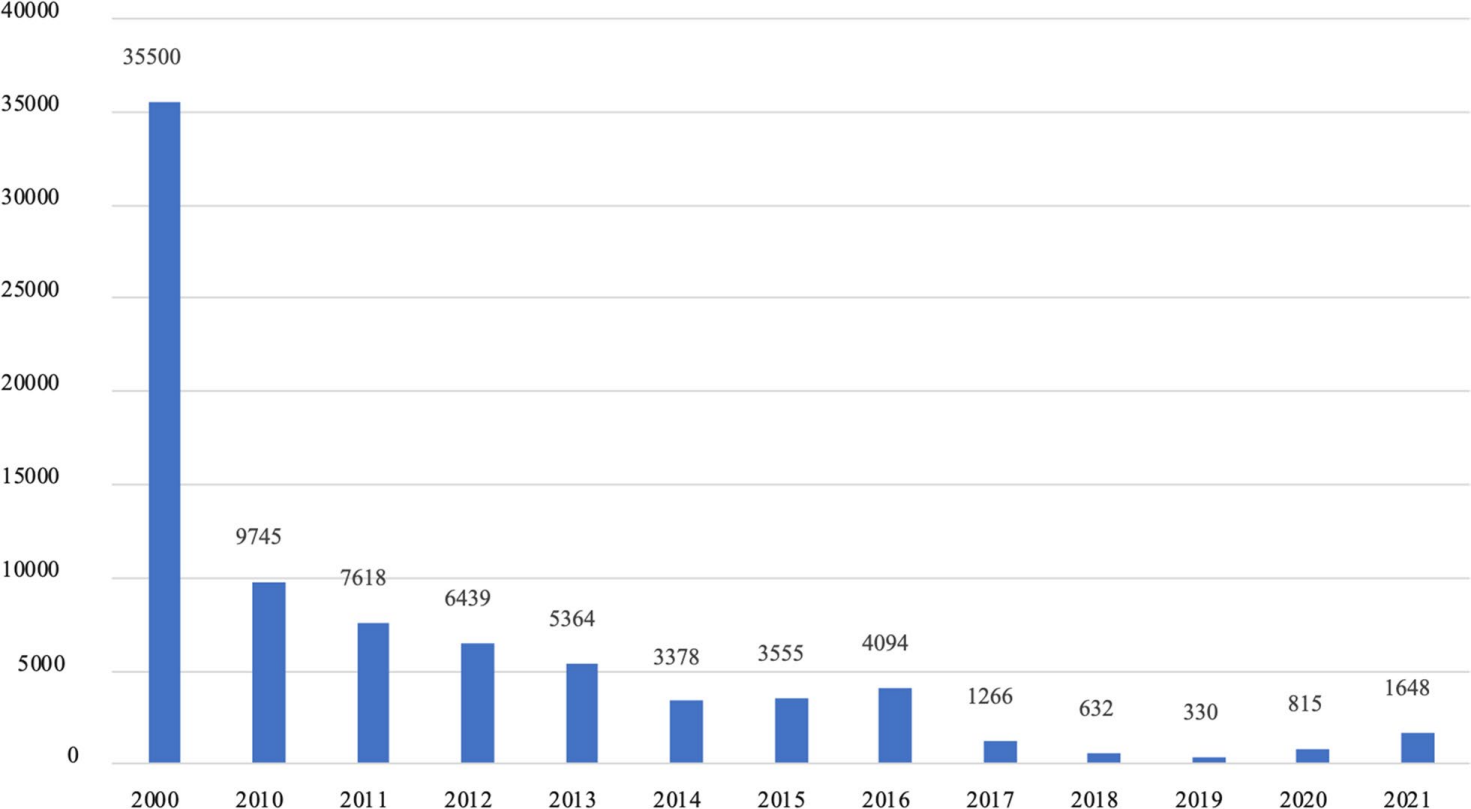
Number of malaria cases per year in Honduras, 2000, 2010–2021

Central America reported approximately 36,000 cases of malaria in 2020, of which 88% are contributed by Nicaragua [5]. Nicaragua shares with Honduras an ecological and anthropological region called La Moskitia, which contributes to the largest number of malaria cases in both countries. Honduras reported more than 1600 cases in 2021, and 97% were from La Moskitia, in the department of Gracias a Dios (Personal communication by the Panamerican Health Organization Office, Honduras). This represents a reduction of more than 95% of cases compared to the year 2000 (Fig. 1). These data classifies Honduras as a country of low endemicity, including it in the list of the 35 countries that have set themselves the goal of eliminating autochthonous transmission of cases by 2030 [6].

Submicroscopic malaria is defined as a low-density *Plasmodium* infection that can only be detected using molecular methods rather than microscopic analysis [7], that is, infections that cannot be detected by LM or a rapid diagnostic test. When transmission rates are reduced in an endemic area, the number of people infected with submicroscopic levels of parasitaemia, as well as asymptomatic cases, tends to increase [8–11]. As a result, diagnostic methods reach their detection limits and become ineffective in detecting submicroscopic and subclinical infections [12, 13]. This scenario is one of the most important challenges for countries that aspire to eliminate malaria, since undiagnosed individuals become reservoirs of the parasite, contributing to low-grade transmission [14, 15]. Therefore, the WHO warns that when there are few cases of malaria at the national or subnational level, national malaria programmes must be adjusted to complete the final phase of elimination [16]. Consequently, more sensitive methods to detect submicroscopic and asymptomatic infections are essential to identify potential reservoirs of transmission and obtain an accurate assessment of malaria epidemiology in low-endemicity areas with the goal of malaria elimination.

After several decades of fighting malaria, Honduras faces the challenge of eliminating malaria in the next 8 years, with transmission restricted to a few municipalities and a low endemicity setting. For this reason, this study aimed to estimate the contribution of submicroscopic carriers of malaria parasites in the Honduran Moskitia region in a symptomatic population using a highly sensitive molecular method.

## Methods

### Study design, setting, and participants

**Fig. 2:**
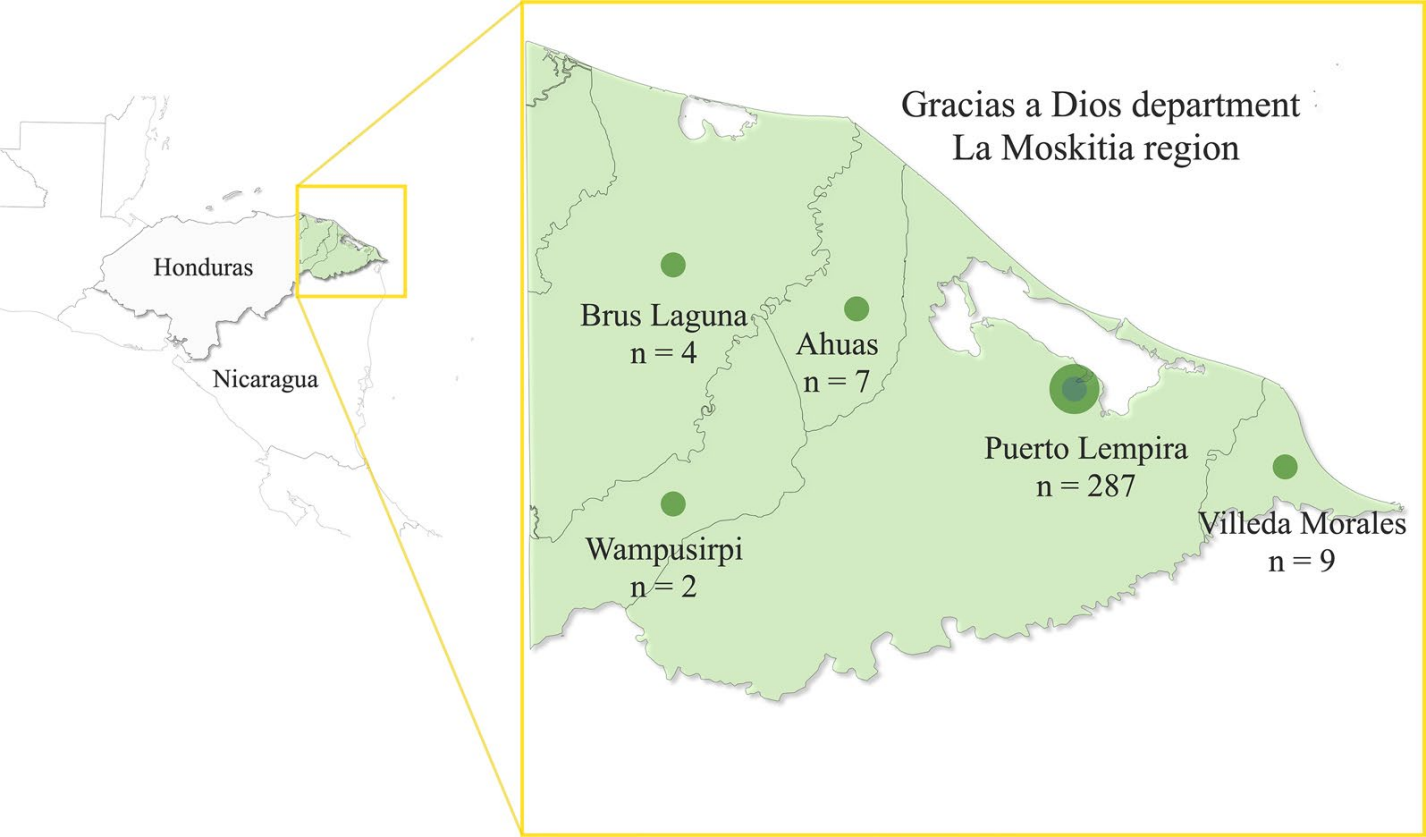
Map of Honduras shows in green the Gracias a Dios department (La Moskitia region) and the municipalities where the participants resided

This was a cross-sectional study that evaluated febrile patients who attended the Puerto Lempira hospital, in Gracias a Dios, in the Honduran region called “La Moskitia”. Samples were collected during 2021 and from January to August 2022. Gracias a Dios is the easternmost department of the country, bordering Nicaragua, characterized by geographic isolation and lack of land communication with the rest of the territory (Fig. 2). Due to isolation and historical and cultural circumstances, the population of La Moskitia lives in conditions of limited socioeconomic development, without adequate access to health services, and low educational levels. La Moskitia accounts for about 98% of malaria cases in Honduras, and currently, the number of cases due to *Plasmodium vivax* reaches 62% while 36% are due to *Plasmodium falciparum*, with 1.7% of mixed infections (National Malaria Surveillance Laboratory, Health Ministry, Honduras; pers. commun.).

Blood samples were collected on the same day as the medical consultation. The demographic data of the patients (age, sex, and municipality of residence) were recorded together with the clinical history. Most of the patients resided in the municipality of Puerto Lempira (92.88%), and the rest came from four other municipalities in the department (Fig. 2). Febrile patients of both sexes and of all ages were recruited. There were no exclusion criteria. The sample size for a low transmission setting was calculated assuming a sensitivity of at least 55%, a specificity of at least 85%, a malaria prevalence of 33.3%, a relative precision of 12%, and 80% power [17]. These criteria yielded a minimum required sample size of 199 subjects.

### Microscopic diagnosis

**Fig. 3:**
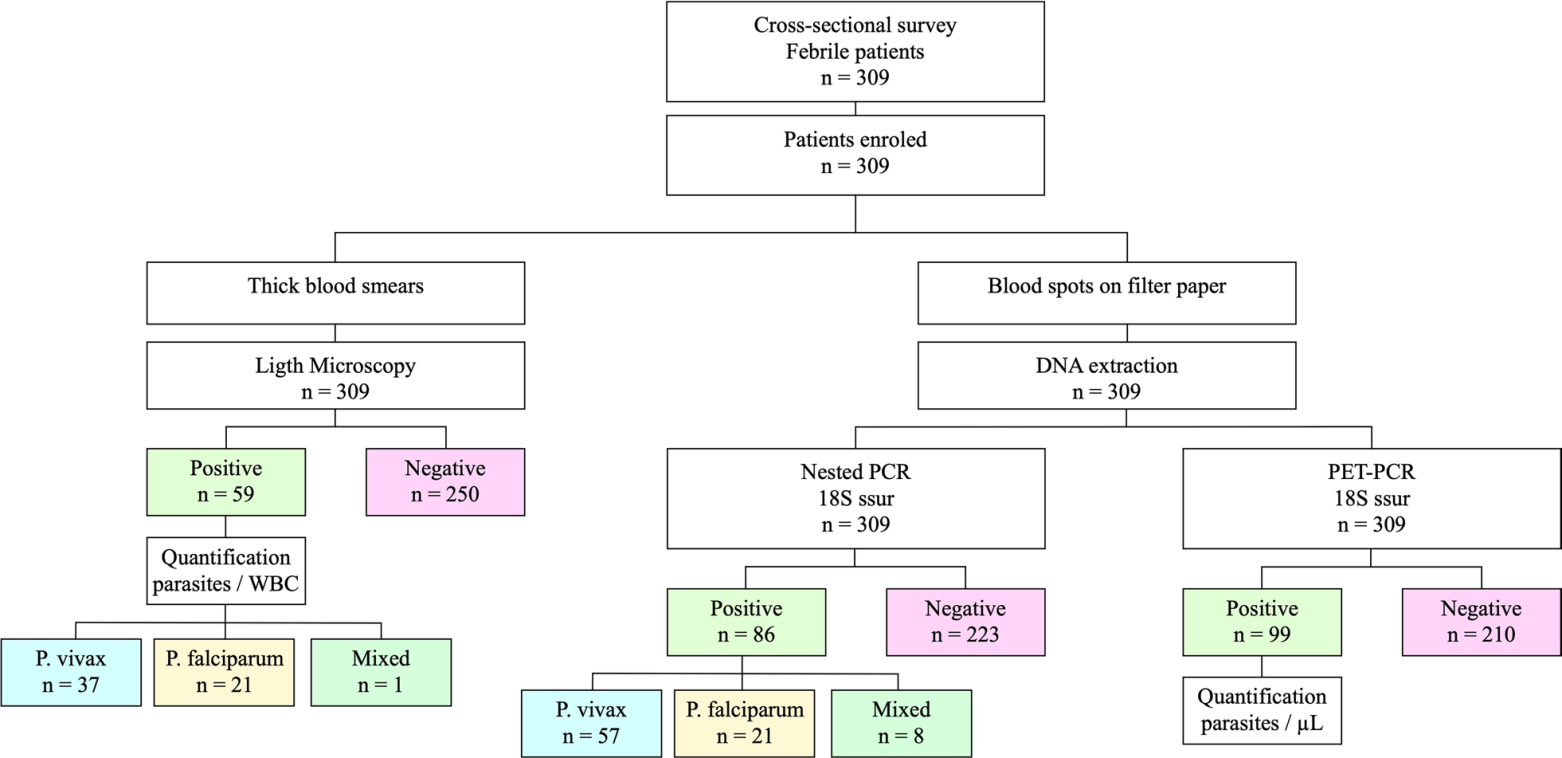
Schematic flow chart showing the number of participants and diagnostic tests

After the medical examination of the patients, the clinical laboratory personnel collected blood samples in tubes with EDTA anticoagulant. In accordance with national malaria guidelines, thick and thin blood smears were prepared for parasitological analysis [18]. Slides were examined within hours of sampling. An expert microscopist observed a maximum of 500 microscopic fields at 100X magnification before reporting the slides as negative. Parasite density was estimated using a quantitative approach in those smears positive for *P. vivax* and/or *P. falciparum*, reporting the total number of sexual and asexual stages per 200 leukocytes. Parasite density was classified as high, moderate, or low, according to parameters established by Alger et al*.* [19]. Patients with a microscopic diagnosis of malaria were treated with chloroquine and primaquine according to national guidelines. Figure 3 shows the workflow used in this study.

### DNA extraction

Two or three drops of blood from each participant were used to impregnate Whatman No. 3 filter paper to preserve the DNA until its subsequent extraction in the city of Tegucigalpa. The samples were placed in sealed plastic bags with desiccant and stored for up to four months. Three circles of 10 mm^2^ each were cut from paper impregnated with blood for DNA extraction. Disks were immersed in 200 µL of 1% saponin, vortexed, and incubated at 4 °C overnight. The next day, samples were washed four times with PBS and then exposed to a 5% Chelex-100 suspension (Bio-Rad, Hercules, CA, USA). Tubes were incubated at 56 °C for 15 min and then at 100 °C for 10 min. Tubes were centrifuged for 5 min at 13,000 rpm and the DNA was recovered from the supernatant and stored at − 20 °C for later analysis. Negative extraction controls were included.

### Nested PCR

A segment of the 18S ribosomal gene of *Plasmodium* spp. was amplified using the nested PCR (nPCR) technique described by Singh et al. [20] with some modifications. Both reactions (first and second round) were carried out in a 50 µL volume containing 25 µL of 2X Taq polymerase master mix (Promega Corp. Madison, WI, USA) and 2 µL of each primer 10 µM (Table 1). The first reaction included 11 µL of nuclease-free water and 10 µL of DNA. The second reaction included 20 µL of nuclease-free water and 1 µL of the PCR product of the first reaction.

**Table 1:** List of primers used for amplification reactions, nucleotide sequences, annealing temperatures, and amplicon sizes

Reaction	Primer	Sequence (5′–3′)	Annealing temperature (ºC)	Product size (bp)
First PCR for *Plasmodium* spp*.*	rPLU1	TCA AAG ATT AAG CCA TGC AAG TGA	55	
	rPLU5	CCT GTT GTT GCC TTA AAC TYC		
Second PCR for *Plasmodium* spp*.*	rPLU3	TTT YTA TAA GGA TAA CTA CGG AAA AGC TGT	62	240
	rPLU4	TAC CCG TCA TAG CCA TGT TAG GCC AAT ACC		
PCR for* P. vivax*	rVIV1	CGC TTC TAG CTT AAT CCA CAT AAC TGA TAC	58	117
	rVIV2	ACT TCC AAG CCG AAG CAA AGA AAG TCC TTA		
PCR for* P. falciparum*	rFAL1	TTA AAC TGG TTT GGG AAA ACC AAA TAT ATT	58	205
	rFAL2	ACA CAA TGA ACT CAA TCA TGA CTA CCC GTC		
PET-PCR for *Plasmodium* spp*.*	Genus forward	GGC CTA ACA TGG CTA TGA CG	63	91
	Labeled–Genus reverse	6FAM- agg cgc ata gcg cct ggC TGC CTT CCT TAG ATG TGG TAG CT		

Negative samples were recorded as negative after the first result. Positive samples were confirmed by a new amplification. If a discordant result was detected between the two amplifications or between the nPCR and the light microscopy (LM), samples were amplified a third time from new DNA extraction. The result was settled by means of two concordant tests. The samples with a final positive result for malaria were analysed to determine the species of the parasite. Two separate reactions were carried out in a final volume of 25 µL containing 12.5 µL of 2X Taq polymerase master mix, 1 µL of each primer (10 µM) (Table 1), 9.5 µL of nuclease-free water, and 1 µL of the product of the first PCR.

Parasite detection by nPCR was blinded to the result obtained by LM, and once the first PCR result was obtained, it was compared with that of the LM to decide whether to repeat the amplification or not.

All reactions (for genus and species) were carried out by an initial denaturation at 94 ºC for 4 min, 35 cycles of 94 ºC for 30 s, annealing temperature for 60 s (Table 1), and 72 ºC for 60 s, with a final extension at 72 ºC for 4 min. Products were visualized by 2% agarose gel electrophoresis with ethidium bromide. Positive and negative controls were included in each set of reactions.

### PET-PCR

The samples were tested in duplicate using a photo-induced electron transfer PCR (PET-PCR) in order to detect *Plasmodium* infections and quantify the number of parasites per µL of blood using absolute quantification of the 18 srRNA gene [21–24]. The parasite genome was detected by amplifying a conserved segment of the 18S ribosomal gene in the four *Plasmodium* species. The reaction was carried out in a volume of 20 µL containing 10 µL of Go Taq^®^ Probe qPCR Master Mix (Promega Corp. Madison, WI, USA), 0.5 µL of each primer (10 µM) (Table 1), 4 µL of nuclease-free water, and 5 µL of DNA. Reactions were run on a Mic qPCR Cycler (Bio Molecular Systems, Brisbane, Australia) and the results were visualized in the Mic qPCR Cycler Software. The amplification conditions for both genus and species detection were 95 ºC for 15 min, 45 cycles at 95 ºC for 20 s, 63 ºC for 40 s, and 72 ºC for 30 s. The correct fluorescence channel was selected for the labelled primer (6FAM). A cycle threshold (Ct) of 40 or below was used to consider samples as positive. Samples with a Ct equal to or less than 40 and a replica with a Ct greater than 40, but less than 2-digit deviation from the positive result were considered positive. The parasite species were not assessed by PET-PCR in this study.

To quantify the parasitaemia, two reference standard curves were included. The first reference curve was prepared with a serial dilution of a well-quantified *P. falciparum* strain 3D7 containing 100,000 parasites per µL. Using this standard curve, the number of parasites/µl present in the sample was estimated based on the Ct value. The mass of DNA equivalent to one *Plasmodium* was not determined because it was not required for the analysis.

The second reference curve included serial dilutions of the plasmid pMG-Amp in which a partial sequence of 200 nucleotides of the *Plasmodium* 18S ribosomal gene was cloned. Both standard curves established the technique's detection limit and determined the samples' parasitaemia.

### Statistical analyses

Sensitivity, specificity, positive (PPV), and negative (NPV) predictive values were calculated for LM and nPCR compared to PET-PCR. Furthermore, these values were also calculated for LM relative to nPCR. Sensitivity was calculated as the ratio of true positives to total positives multiplied by 100. Specificity was calculated as the ratio of true negatives to total negatives multiplied by 100. 95% confidence intervals were calculated for sensitivity and specificity. PPV was calculated as follows: true positives/(true positives + false positives) * 100; and NPV as: true negatives/(true negatives + false negatives) * 100. Diagnostic accuracy was calculated as: (true positives + true negatives)/(true positives + true negatives + false positives + false negatives) * 100 [25]. The total number of samples was those on which all three assays were successfully performed. McNemar's test was calculated between LM and nPCR results.

Receiver operating curve (ROC) analysis and areas under the curve (AUC) were carried out using a pROC library implemented in R to assess diagnostic accuracy and to compare the diagnostic performance of LM, nPCR, and PET-PCR. AUC was interpreted as follows: 0.9–1.0, excellent; 0.8–0.9, very good; 0.7–0.8, good; 0.6–0.7, sufficient; 0.5–0.6, bad; < 0.5, test not useful [26].

The Cohen's kappa coefficient of agreement between LM and nPCR and PET-PCR was also computed as k = p_o_–p_e_/1–p_e_, where p_o_ was the relative observed agreement among assays, and p_e_ was the hypothetical probability of chance agreement. In addition, the kappa index between LM and nested PCR was calculated using nPCR as a reference. McHugh's table was used to interpret kappa values [27].

### Ethical approval

The study was conducted according to the guidelines of the Declaration of Helsinki and approved by the ethics committee (CEI-MEIZ) of the National Autonomous University of Honduras (UNAH) under protocol number 03-2020. The patients or their legal guardians were informed of the objectives of the study and signed an informed consent form before collecting the blood samples.

## Results

### Characteristics of the population

The study included a total of 309 febrile participants recruited using a routine passive surveillance approach. The patients resided in five municipalities of the department of Gracias a Dios: 287 from Puerto Lempira, 9 from Villeda Morales, 7 from Ahuas, 4 from Brus Laguna, and 2 from Wampusirpi (Fig. 2). Most of the participants (67.96%) were female, and 15.53% of the patients were under 5 years old. The average age of the participants was 23.1 years, while the average age of patients with positive PET-PCR results was 21.91 years.

### Malaria detection

The number of positive cases of malaria detected by the three methods is described in Table 2. Microscopy diagnosed 59 cases, while nPCR and PET-PCR detected 86 and 99 positive cases, respectively. The percentage of *P. vivax*/*P. falciparum* infections were 62.7%/35.6% by LM, and 66.3%/24.4% by nPCR. Nested PCR detected 9.3% of mixed infections, compared to only 1.7% detected by LM.

**Table 2:** Number of positive and negative samples for malaria according to three diagnostic assays and parasite species identification

Method	Positive samples (%)	Negative samples (%)	*P. vivax* (%)	*P. falciparum* (%)	Mixed (%)
Microscopy	59 (19.09)	250 (80.9)	37 (62.7)	21 (35.6)	1 (1.7)
Nested PCR	86 (27.83)	223 (72.17)	56 (65.1)	22 (25.6)	8 (9.3)
PET-PCR	99 (32.04)	210 (67.96)	Not available (N/A)	N/A	N/A

In positive cases by LM, the number of parasites per 200 leukocytes was determined, and the results were classified into three groups. The cases with high parasitaemia were 55.9%, while 27.1% showed moderate parasitaemia, and 16.7% showed low parasitaemia.

Table 3 describes the percentages of concordance between LM and nPCR in terms of the identification of parasite species. LM was correct in 33 of 36 (91.7%) *P. vivax* infections and in 14 of 15 (93.3%) *P. falciparum* infections compared to nPCR. However, LM was only able to detect 1 of 8 (12.5%) mixed infections.

**Table 3:** Concordance in the diagnosis of *Plasmodium* species between microscopy and nested PCR

	Light Microscopy				
nPCR	*P. vivax* (%)	*P. falciparum* (%)	Mixed (%)	Negative (%)	Total
*P. vivax* (%)	33 (10.68)	3 (0.97)	0	20 (6.47)	56 (18.12)
*P. falciparum* (%)	1 (0.32)	14 (4.53)	0	7 (2.27)	22 (7.12)
Mixed (%)	3 (0.97)	4 (1.29)	1 (0.32)	0	8 (2.6)
Negative (%)	0	0	0	223 (72.17)	223 (72.17)
Total	37 (11.97)	21 (6.8)	1 (0.32)	250 (80.9)	309 (100)

### Comparison of diagnostic test results

The performance of the diagnostics methods (LM, nPCR, and PET-PCR) was compared. The sensitivity of LM and nPCR compared to PET-PCR was 59.6% and 80.8%, respectively. On the other hand, the specificity of both methods was 100% and 97.14%. Similarly, the sensitivity and specificity of LM for nPCR were 67.4% and 99.56%, respectively. The rest of the diagnostic statistics are shown in Table 4. Statistically significant differences were found between the sensitivity of LM and nPCR, according to McNemar’s test (p < 0.001).

**Table 4:** Microscopy and nested PCR performance values in relation to PET-PCR

Diagnostic performance	Light Microscopy	Nested PCR
Sensitivity [95% CI]	59.6% [54.7–64.5%]	80.8% [72.05–89.54%]
Specificity [95% CI]	100% [88.85–111.14%]	97.14% [86.31–107.96%]
PPV	100%	93.02%
NPV	84%	93.15%
Accuracy	87.06%	91.9%
Kappa index	0.6672	0.8075

**Fig. 4:**
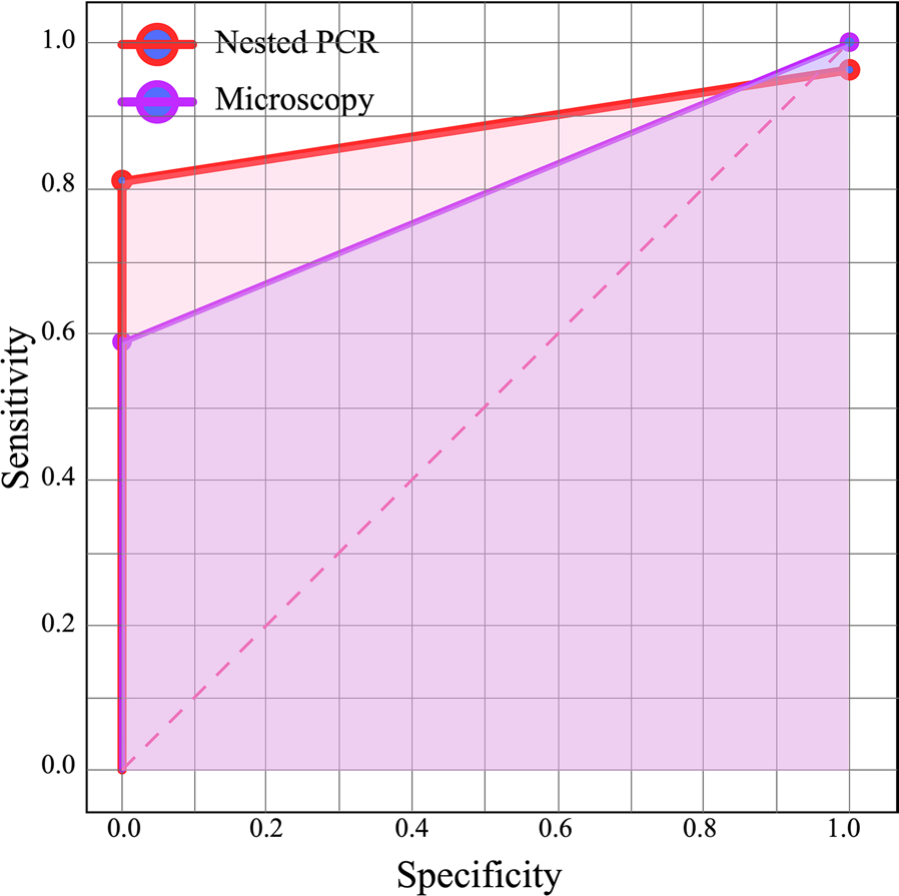
ROC curves for light microscopy and nested PCR compared with PET-PCR. Nested PCR showed an AUC of 89% and LM an AUC of 79.8%

Concordance, relative to PET-PCR, expressed as Cohen's kappa coefficient was 66.7% and 80.8% for LM and nPCR, respectively. ROC analysis showed that the area under de curve (AUC) was 79.8% (good) for LM and 89.0% (very good) for nPCR (Fig. 4). Fifty-eight cases (18.77%) were positive by the three methods.

### Parasitaemia quantification

The number of parasites per µL of blood was determined by LM and PET-PCR. An average of 4395 parasites/µL (range 32–17840 parasites/µL) was detected by LM (n = 59). According to PET-PCR (n = 99) the average number of parasites/µL was 776.4 [0.072–6737] and the average Ct of all the positive samples was 32.55 [23.82–40]. The average number of parasites/µL for the population under 15 years was 529 [0.1–3135] and an average Ct of 36.7.

The average Ct was calculated for three subgroups. The cases with low parasitaemia by LM showed an average Ct of 31.9 (range 27.2–38.0). Those with moderate parasitaemia showed an average Ct of 28.8 (range 27.52–32.8), and an average Ct of 27.2 (range 24.1 28.1) was the result for cases with high parasitaemia.

In addition, the average Ct, and the number of parasites per µL were calculated in 40 negative samples by LM but positive by PET-PCR, as well as in 19 negative samples by nPCR and positive by PET-PCR (Table 5). A similar analysis was performed on positive samples only by PET-PCR and negative for any other method (Table 5).

**Table 5:** Average Ct and parasitaemia in positive samples by PET-PCR and negative by microscopy and/or nPCR

	Light microscopy negative	nPCR negative	LM negative / nPCR negative
Average Ct	37.91 [33.45–41.46]	35.13 [26.0–43.1]	39.04 [35.9–41.5]
Parasites/µL	0.94 [0.1–18.25]	5.2 [0.1–32.0]	0.30 [0.1–3.4]

Forty negative cases by LM had discordant results by PET-PCR (Ct ≥ 40). Sixteen negative cases by LM had two positive results by PET-PCR. Nineteen nPCR-negative cases had one positive and one negative result by PET-PCR, while seven cases had two PET-PCR positive results. In total, 83 concordant results were recorded between both PET-PCR replicates, while 16 showed discrepant results (one positive and one negative). The average Ct of the 83 concordant reactions was 30.5, while the average Ct of the 16 discordant reactions was 39.53. Fifteen cases were recorded in which the PET-PCR showed a negative result in both replicates, with a Ct reading greater than 40 and less than 43.1. The PET-PCR detection limit was established at 0.2 parasites/µL for a Ct equal to 40.

## Discussion

In this study, the diagnostic performance of two methods (LM and nPCR) to detect malaria in a low endemicity setting was compared [5] using a high sensitivity technique (PET-PCR) [22]. LM is the method commonly used for passive surveillance in Honduras, although rapid diagnostic tests are also widely used when conditions are not favorable for microscopy, or for active and reactive case searches [18]. Also, the Honduran Ministry of Health uses a molecular method (nPCR) [20] for evaluating the quality of malaria diagnosis in two local reference laboratories.

Microscopy was able to detect only 59 positive cases (19%), with a sensitivity of less than 60%, while molecular methods (nPCR and PET-PCR) proved to be much more sensitive. Because of LM's low sensitivity, 40 cases were misdiagnosed as false negatives, resulting in these patients not receiving adequate treatment. Untreated infections maintain transmission in the region, hindering the achievement of elimination goals. The presence of submicroscopic infections supports the arguments of those who advocate the implementation of more radical measures such as massive or focal administration of treatments [28, 29].

Although microscopy is still considered the gold standard diagnostic method in many countries, it has repeatedly been shown to have poor sensitivity relative to molecular methods [24, 30–32], especially when parasite densities are low [13, 33, 34]. In a study conducted among febrile patients from the Honduran Moskitia, the sensitivity of LM and a rapid diagnostic test based on haemozoin detection were compared against a molecular method, revealing that the sensitivity of LM was less than 65% [35]. In a study conducted in the Peruvian Amazon, a molecular method detected nearly sevenfold and 25-fold higher prevalence than LM for *P. vivax* and *P. falciparum* infections, respectively, when packed red blood cells were used as starting material for quantitative PCR [13]. These and other studies have shown that a high burden of submicroscopic infections is an increasingly common scenario in different geographic regions [12, 28, 36–38].

In addition to the low sensitivity demonstrated by LM, an added problem of this approach is the misdiagnosis of parasite species and the inability to diagnose mixed infections. Patients with *P. vivax* infections and mixed infections must receive a different treatment of primaquine (0.25 mg/kg for 14 days or 0.5 mg/kg for 7 days) compared to *P. falciparum* infections (0.75 mg/kg in a single dose), consequently, a misdiagnosis could prevent the elimination of all hepatic forms of *P. vivax* [18].

In this study, LM showed that 17% of the positive cases had low parasitaemia. The average Ct of the samples with low parasitaemia by LM was 31.9, while the average Ct of the negative samples by LM but positive by PET-PCR was considerably higher (Ct = 37.91). Likewise, the average Ct of the samples positive only by PET-PCR but negative by LM or nPCR was 39.04, bordering the established cut-off point of 40.

The quantification of parasites in peripheral blood is limited by the sensitivity of the diagnostic method and will always be an approximation. According to LM, the average number of parasites/μL for the LM-positive samples was 4400 [32–17840], while PET-PCR determined that the average was 776 [0.1–6737]. This confirms the inaccuracy of LM when quantifying parasitaemia at low levels [39], and that the molecular method shows greater sensitivity to detecting submicroscopic infections. This result supports what was reported by the WHO: In general, a good microscopist detects up to 50 parasites/μL of blood, and an expert microscopist can detect up to 20 parasites/μL, while a quantitative PCR can offer a limit of detection of up to 0.02 parasites/μL [40].

Several reports already use the novel fluorogenic self-quenching photoinduced electron transfer (PET-PCR) primers to quantify the parasitaemia of malarial infections. When this technique was described, the authors suggested using 2 µL of DNA to obtain a detection limit of 3.2–5.8 parasites/µL [22]. Subsequently, the starting DNA volume has been increased to 5 µL [23, 24, 41–45] to improve the sensitivity of the method. In this study, 5 µL of DNA and a cycle threshold (Ct) below 40 were used, as recommended by most reports [21, 24, 41, 42, 44], although some authors have used 40.5 or 41 [22, 23]. With a Ct below 40, PET-PCR was able to detect less than 0.2 parasite/µL of blood, which meets the WHO’s Evidence Review Group recommendation of at least 2 parasites/µL for molecular assays [40]. However, 15 cases showed a Ct above 40 in both replicates, which could be real infections with very low parasitaemia. If at least some of these 15 cases as true positives, the sensitivity of LM would be even lower.

This result highlights the high number of febrile cases with submicroscopic infections in the Honduran Moskitia, an occurrence that has been commonly observed in low-endemicity settings [10, 46]. This phenomenon can be explained by the advantage that the less virulent strains of the parasite would prevail over the more prolific strains, which would be more likely to be detected and eliminated by the treatment [46]. A second hypothesis attributes the phenomenon to the increase in protective immunity among the population due to the decrease in the antigenic diversity of the circulating strains of the parasite [10]. Recently, a bottleneck effect in the population of *P. falciparum* strains circulating in Honduras has been demonstrated, lending support to the hypothesis of premunition as the cause of the low parasitaemias observed in this study [47].

There are two limitations to this study. The species of the parasite was not identified using PET-PCR, and the participants' febrile state could have been caused by other clinical conditions other than malaria.

## Conclusion

This study demonstrates that many febrile patients are not properly diagnosed due to the low levels of parasites circulating in the blood. Presumably, an even greater number of individuals suffer from asymptomatic malaria infections in La Moskitia. Both groups of individuals, febrile patients with submicroscopic infections and asymptomatic carriers, do not receive treatment, remaining as reservoirs and hindering the goal of malaria elimination. Further studies should include large-scale surveys of asymptomatic people using highly sensitive methods such as PET-PCR to better understand the real malaria situation in Honduras and reorient control programmes toward elimination.

## Abbreviations

AUC: Area under curve
Ct: Cycle threshold
EDTA: Ethylenediaminetetraacetic acid
LM: Light microscopy
nPCR: Nested PCR
NPV: Negative predictive value
PBS: Phosphate buffered saline
PET-PCR: PCR based on photo-induced electron transfer fluorogenic primers
PPV: Positive predictive value
qPCR: Quantitative PCR
ROC: Receiver operating curve
RPM: Revolutions per minute
WHO: World Health Organization
6FAM: 6-Carboxyfluorescein
